# The effect of primary total knee arthroplasty on the incidence of falls and balance-related functions in patients with osteoarthritis

**DOI:** 10.1038/s41598-017-16867-4

**Published:** 2017-11-29

**Authors:** Hai-bo Si, Yi Zeng, Jian Zhong, Zong-ke Zhou, Yan-rong Lu, Jing-qiu Cheng, Ning Ning, Bin Shen

**Affiliations:** 1 Department of Orthopaedics, West China Hospital, Sichuan University, Chengdu, 610041 China; 2 Key Laboratory of Transplant Engineering and Immunology, West China Hospital, Sichuan University, Chengdu, 610041 China

## Abstract

Knee osteoarthritis (OA) is an established risk factor for falls and balance impairment. This study investigated the incidence of falls, balance-related outcomes and risk factors for falls before and after primary total knee arthroplasty (TKA). Three hundred seventy-six OA patients scheduled to undergo TKA were included. Falls data within the preoperative, first postoperative and second postoperative years were collected, balance-related functions were assessed using the Assessment of Quality of Life (AQoL), WOMAC, Falls Efficacy Scale International (FES-I), Activities-specific Balance Confidence (ABC), knee extension strength, Berg Balance Scale (BBS) and Timed Up and Go (TUG) before surgery and 1 and 2 years after surgery. Compared with preoperative values, the incidence of falls significantly decreased (14.89%, 6.23% and 3.14% within the preoperative, first postoperative and second postoperative years, respectively) and the AQoL, WOMAC, FES-I, ABC, knee extension strength, BBS and TUG significantly improved after TKA. Logistic regression analysis revealed that Kellgren-Lawrence grade ≥ 3 of the contralateral knee was an independent risk factor for falls before and after TKA. Conclusively, primary TKA is associated with a reduced incidence of falls and improved balance-related functions, and the contralateral knee should be considered in the design of fall-prevention strategies in patients with OA.

## Introduction

Falls are among the most common causes of injury and hospitalization in the elderly, and an estimated 1/3 of people over the age of 65 fall each year^1^. The reported annual incidence of falls among older Chinese community-dwelling people ranges from 11% to 34%, and the annual incidence of more than one fall is 4% to 5%^2^. Balance is a complex function that requires the integration of sensory information of the position of the body and the ability to make proper motor responses to body movement^3^. Balance is essential for maintaining postural stability while performing daily activities^4^, and loss of balance is a common cause of falls in older people^5,6^.

Osteoarthritis (OA) is a widely prevalent and age-associated joint disorder, and it is an important risk factor for falls. More than 50% of all OA patients and 64% of female OA patients report falling within one year^5,7–9^. Approximately 60% to 80% of patients with knee OA report joint instability^10,11^. No effective treatments are currently available to prevent or reverse OA progression^12^, but total knee arthroplasty (TKA) is a common surgical intervention for treating end-stage knee OA. TKA aims to relieve pain, restore loco-motor functions, correct deformities and improve quality of life^13^. Patients with knee OA undergoing TKA often present with residual functional deficits, and one important area of concern is balance impairments, which increase the risk of falls in these patients^14–17^. The incidence of falls is as high as 7% to 40% after TKA^18–20^.

An understanding of the issues associated with the recovery of balance-related functions following TKA may ultimately enhance the design of fall-prevention programmes supported by scientific evidence^3^. Previous studies assessed balance-related functions in OA patients only during early stages after TKA at 1 year or less in small samples, and the results were inconsistent and controversial^3,5,21^. OA often involves bilateral knees^22^, and previous studies demonstrated that the pain and knee extension strength of the contralateral knee were associated with knee functions after TKA^22,23^. However, few studies investigated the impact of the OA status of the contralateral knee on the incidence of falls after primary TKA. We hypothesize that the annual incidence of falls and balance-related functions will improve after primary TKA, and the OA status of the contralateral knee, age and sex are risk factors for falls before and after TKA in OA patients. This study investigated the following specific issues: (1) the annual incidence of falls before and after primary TKA; (2) the effect of primary TKA on balance-related outcomes, such as fear of falling, balance confidence and physical functions; and (3) the possible risk factors for falls before and after primary TKA in patients with knee OA.

## Methods

### Patients

Consecutive patients who were scheduled for primary unilateral TKA at the Department of Orthopaedics, West China Hospital, Sichuan University were assessed from August 2013 to December 2014 and invited to participate if they met the following eligibility criteria: (1) diagnosis of end-stage primary knee OA with no previous surgery on either knee; (2) absence of uncontrolled systemic diseases, neurological, cardiac, psychiatric disorders, or other medical conditions that would significantly compromise physical functions (e.g., stroke); and (3) sufficient language skills to communicate, the ability to follow verbal instructions, and no memory problems. Patients were excluded for any of the following reasons: (1) if they did not understand the nature of the study; (2) if they underwent TKA due to non-OA diseases, such as rheumatoid arthritis (RA) and traumatic injury; or (3) if they underwent reoperation of the replaced knee or received contralateral knee arthroplasty during the follow-up period.

Ethics approval was obtained from the Ethics Committee of West China Hospital, Sichuan University, and all procedures were performed according to the Declaration of Helsinki. All participants were informed of the nature of the study and signed a written informed consent before participation.

### Total knee arthroplasty

The same surgical team performed all surgeries using a standard medial parapatellar approach in the same laminar air flow operating room, and a posterior stabilized knee prosthesis system (DePuy, New Jersey, USA) with a mobile or fixed bearing was used in all patients. A pneumatic tourniquet was used in all patients, which was inflated before skin incision and released after prosthesis placement, and a suction drainage was indwelled before suturing and removed the first morning after surgery. All patients received similar perioperative physiotherapy, such as enhanced perioperative education, muscle strengthening and gait re-education before surgery, flexion-extension of the ankle joints and straight leg raising immediately after anaesthesia awakening, and flexion-extension of the operated knee, muscle strengthening and gait re-education beginning on the first postoperative day (POD). Fall-prevention strategies, including an enhanced explanation of the hazards and risk factors of falls to enhance patient consciousness of fall-prevention, correcting high-risk activities, introducing methods to avoid falling during daily activities, and guided preoperative and postoperative functional exercises were performed during hospitalization (face to face) and after discharge (quarterly by telephone and at each follow-up visit). The surgical characteristics, including incision length, operative time (from the skin incision to the end of skin suture) and prosthesis type (mobile or fixed) were recorded.

### Self-reported measures

After being recruited to the study, participants completed a series of self-reported questionnaires and physical performance tests for falls, quality of life, fear of falling, balance confidence and physical functions one day before surgery (T0), and 1 year (T1) and 2 years (T2) after surgery. A fall was defined as unintentionally coming to rest on the ground or at some other lower level, not as a result of a major intrinsic event, such as faint or stroke^3^. Preoperative fall data within the previous 1 year were collected at T0, and postoperative fall data were collected quarterly by telephone and confirmed at the yearly follow-up visit. Quality of life was measured using the self-reported Assessment of Quality of Life (AQoL), which is composed of five domains, including illness, independent living, social relationships, physical senses and psychological well-being, with three items per domain^24^. The utility scores for each dimension and an overall utility score from 0 to 1 (0 representing the worst health and 1 representing perfect health) were collected.

The Short Falls Efficacy Scale International (FES-I) questionnaire was used to evaluate the fear of falling^25^. The FES-I consists of seven items (score 1–4 for each) that evaluate the participant’s level of concern of the possibility of falling when performing certain daily activities, and the total score ranges from 7 (not concerned) to 28 (severely concerned). The self-reported Activities-specific Balance Confidence (ABC) Scale was used to quantify how confident a person feels that he or she will not lose balance while performing 16 certain daily activities on a scale from 0% (absolutely not confident) to 100% (completely confident)^18^. The total score was averaged across the 16 items, and higher scores indicate greater balance confidence.

Pain, stiffness and physical functions of the knee were also assessed using the Western Ontario and McMaster University Osteoarthritis Index (WOMAC)^26^, which assesses over three domains, including the severity of knee pain during 5 daily activities (range 0–500), stiffness (range 0–200), and physical functions of the lower extremities during 17 daily activities (range 0–1,700). The items were scored using a 100-mm visual analogue scale, where 0 represents no pain, stiffness or difficulty with physical functions, and higher scores represent worse functional health. All three subscales are summed to provide a total WOMAC score (range 0–2,400).

### Physical performance tests

The knee extension strength, Berg Balance Scale (BBS), and Timed Up and Go (TUG) were used to further assess balance-related functions. The knee extension strength was measured via isometric pulling at 90° against padded straps attached to an electronic dynamometer (HANDPI^TM^, Zhejiang, China). The highest value (kg) of the three trials was recorded and normalized to the patient’s body height and body mass using the following formula^5^: normalized knee extension strength = measured strength (kg)/[body height (m) × body mass (kg)] × 100. The BBS is a physical assessment of balance comprising a set of 14 balance tasks^27^. Each subset was scored on a 5-level ordinal scale from 0 to 4, yielding a maximum total score of 56. Higher scores indicate better balance, and scores below 45 indicate an increased risk of falls. A change of 4–7 points in the elderly is the estimated minimal detectable change at a 95% confidence level (MDC_95_) that may be objectively determined for a patient^28^. TUG is a test of functional mobility in older people^29^. The patient is observed and timed while getting up from an armchair, walking 3 metres, and returning to their original seated position. Older adults who take longer than 14 sec to complete the TUG have a high fall risk^30^, and the minimal level of detectable change at the 90% confidence level (MDC_90_) is 2.49 sec.^31^. Researchers with rich clinical experience in assessing elderly performed the physical assessments, and the researchers were blinded to the preoperative results upon performing the postoperative assessments.

### Statistical analysis

All statistical analyses were performed using SPSS software (version 22.0, IBM, USA). All continuous variables were assessed for normality of distribution using the Kolmogorov-Smirnov and Shapiro-Wilk tests prior to data analysis. Comparisons of continuous variables between two groups were made using the *t*-test for normally distributed data or the Wilcoxon signed rank sum test for non-normally distributed data. Comparisons of continuous variables between three or more groups were performed using one-way analysis of variance (ANOVA) with Student-Newman-Keuls (SNK) *post hoc* analysis for normally distributed data or the Kruskal-Wallis *H* and Mann-Whitney *U* tests for non-normally distributed data. Comparisons of dichotomous variables were performed using the *Cochran Q* test for repeated measures or the *Chi-square* (*χ* ^2^) test for non-repeated measures. Binary logistic regression was performed to identify independent risk factors for falls, and the general and surgical factors, including age, sex, body mass index, KL grade of the contralateral knee, incision length, operative time and prosthesis type, were included in the model. A *P* value less than 0.05 was considered statistically significant.

## Results

### Subjects

**Figure 1:**
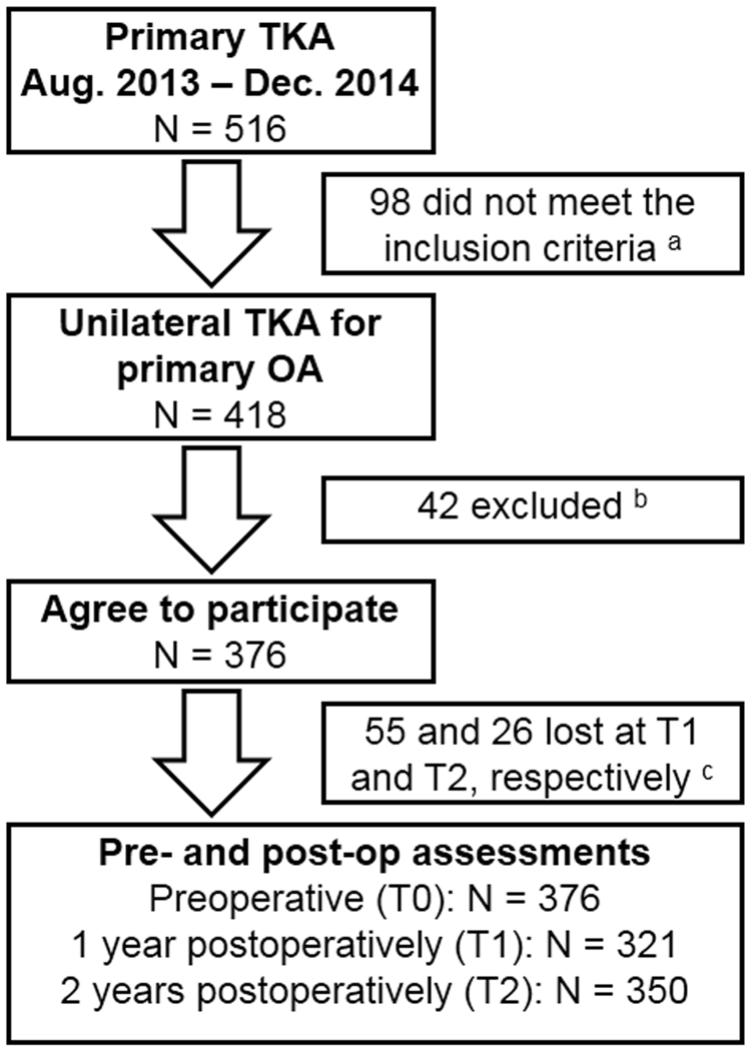
Flow diagram of the study. (**a**) Reasons for exclusion were patients undergoing bilateral TKA or undergoing TKA because of rheumatoid or traumatic arthritis. (**b**) Reasons for exclusion were patients who did not understand the nature of the study, refused to participate, were illiterate, or were physically or mentally unable to complete the questionnaires. (**c**) Patients who were included but were lost by the yearly follow-up because of various reasons, such as traffic or health problems.

Figure 1 provides the full details of the study flow. A total of 376 OA patients (117 males) undergoing primary and unilateral TKA were included preoperatively, with a mean age of 68.88 (47–89) years. A total of 321 (85.37%) and 350 (93.09%) patients were followed-up at T1 and T2, respectively. There were no significant differences in the general and surgical characteristics, including age, gender, body mass index (BMI), KL grade of the contralateral knee, incision length, operative time and prosthesis type, between patients at T0 to T2 (all *Ps* < 0.05).

### Fallers and falls before and after primary TKA

Table 1 shows the details of the pre- and postoperative fallers and falls. A total of 14.89% (56/376) of patients fell, encompassing a total of 101 falls in the last preoperative year. A total of 6.23% (20/321) of patients had a total of 27 falls in the first postoperative year, and 3.14% (11/350) of patients had a total of 14 falls in the second postoperative year. A total of 58.93% (33/56) of the fallers experienced more than one fall in the last preoperative year compared to 25.00% (5/20) and 18.18% (2/11) in the first and second postoperative years, respectively. There were significant reductions in the postoperative number of total fallers (the incidence of falls) and fallers with more than one fall compared to the preoperative values (*Q* = 50.98, *P* < 0.01; *Q* = 46.69, *P* < 0.01, respectively).

**Table 1:** Characteristics of pre- and postoperative fallers and falls.

Group	T0	T1	T2
Patients (N)	376	321	350
Total fallers	56 (14.89%)	20 (6.23%)*	11 (3.14%)*^#^
>1 fall fallers	33 (58.93%)	5 (25.00%)*	2 (18.18%)*
Total falls	101	27	14
Location			
Inside	55	16	6
Outside	33	10	8
Not stated	13	1	0
Injuries			
No injuries	33	10	7
Cuts/bruises	48	13	5
Fractures	3	1	0
Unclassifiable	17	3	2
Hospitalized	6	3	1

T0, before surgery; T1, 1 year after surgery; T2, 2 years after surgery. *Compared with preoperative values, *P* < 0.05; ^#^Compared with T1 values, *P* < 0.05.

A total of 76.00% (38/50, n = 321) and 90.57% (48/53, n = 350) of preoperative fallers did not fall again in the first and second postoperative years, respectively, and 2.95% (8/271, n = 321) and 2.02% (6/297, n = 350) of preoperative non-fallers fell in the first and second postoperative years, respectively. A significant shift in favour of preoperative fallers becoming postoperative non-fallers was observed (*Q* = 78.18, *P* < 0.01). A total of 13.03% (49/376) of patients required an aid to walk independently outside at T0, and this percentage was significantly reduced to 6.85% (22/321) and 3.71% (13/350) at T1 and T2, respectively (*Q* = 58.50, *P* < 0.01).

### Balance-related outcomes before and after primary TKA

Table 2 shows the serial changes in balance-related outcomes. The AQoL, WOMAC (total and subscales), FES-I, ABC, knee extension strength, BBS and TUG improved significantly after primary TKA compared with preoperative values (all *Ps* < 0.01). The AQoL was significantly higher at T2 than at T0 and T1, but no significant difference was found between the scores at T0 and T1. The WOMAC (total and subscales) and FES-I were significantly lower at T1 and T2 than at T0, and the scores were also significantly lower at T2 than at T1. The ABC was significantly higher at T1 and T2 than at T0, and the ABC was also significantly higher at T2 than at T1. Physical performance tests revealed that the knee extension strength and BBS were significantly higher at T1 and T2 than at T0, and the knee extension strength was also significantly higher at T2 than at T1. No significant difference was observed between the BBS at T1 and T2. A total of 61.68% (198/321) and 57.71% (202/350) of patients exhibited increased BBS scores at T1 and T2, respectively, and 45.48% (146/321) and 43.71% (153/350) exceeded the MDC_95_ (4 points) at T1 and T2, respectively. In contrast, the TUG was significantly lower at T1 and T2 than at T0, and the TUG was also significantly lower at T2 than at T1. A total of 68.85% (221/321) and 70.86% (248/350) of these patients exhibited a decrease in TUG at T1 and T2, respectively, and 59.81% (192/321) and 62.00% (217/350) demonstrated an improvement greater than the MDC_90_ (2.49 sec) at T1 and T2, respectively.

**Table 2:** The serial changes in balance-related outcomes (mean ± SD).

	T0	T1	T2	*P*
Patients (N)	376	321	350	
AQoL	0.70 ± 0.11	0.71 ± 0.17	0.75 ± 0.13*^#^	<0.01
WOMAC total	1032.60 ± 347.76	443.53 ± 188.56*	353.36 ± 147.57*^#^	<0.01
Pain	230.74 ± 115.54	114.33 ± 69.88*	93.05 ± 60.77*^#^	<0.01
Stiffness	102.59 ± 51.31	52.25 ± 28.43*	31.39 ± 20.00*^#^	<0.01
Function	699.27 ± 326.55	276.94 ± 166.88*	228.93 ± 130.62*^#^	<0.01
FES-I	15.89 ± 4.72	12.40 ± 3.16*	11.24 ± 2.43*^#^	<0.01
ABC	58.95 ± 15.63	68.10 ± 17.61*	74.70 ± 14.12*^#^	<0.01
Knee extension strength	19.05 ± 9.70	23.06 ± 8.89*	25.45 ± 11.10*^#^	<0.01
BBS	45.69 ± 4.98	48.00 ± 4.93*	48.16 ± 4.65*	<0.01
TUG (sec)	19.31 ± 8.52	13.43 ± 6.04*	12.54 ± 5.37*^#^	<0.01

T0, before surgery; T1, 1 year after surgery; T2, 2 years after surgery; AQoL, Assessment of Quality of Life; WOMAC, Western Ontario and McMaster University Osteoarthritis Index; FES-I, Falls Efficacy Scale International; ABC, Activities-specific Balance Confidence; BBS, Berg Balance Scale; TUG, Timed Up and Go. *Compared with preoperative values, *P* < 0.05; ^#^compared with the values at T1, *P* < 0.05.

### Comparisons between fallers and non-fallers before and after primary TKA

**Figure 2:**
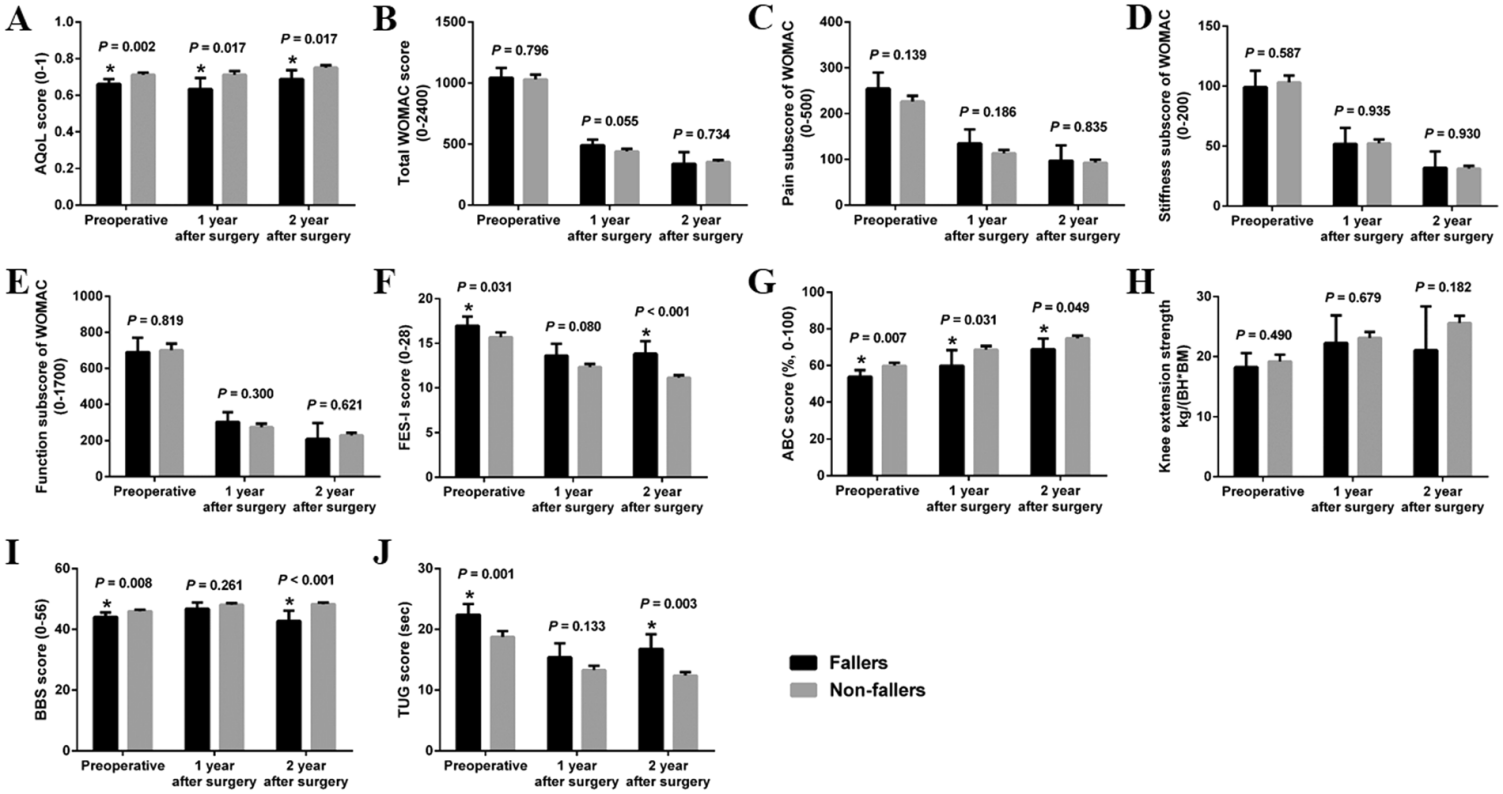
Comparisons of balance-related outcomes between fallers and non-fallers. AQoL, Assessment of Quality of Life; WOMAC, Western Ontario and McMaster University Osteoarthritis Index; FES-I, Falls Efficacy Scale International; ABC, Activities-specific Balance Confidence; BH, Body Height (m); BM, Body Mass (kg); BBS, Berg Balance Scale; TUG, Timed Up and Go. *Compared with non-fallers, *P* < 0.05.

Table 3 shows that the mean age and percentage of female patients were significantly higher in fallers than in non-fallers at T0 and T1, and the KL grade of the contralateral knee was significantly higher in fallers than in non-fallers at T0 to T2 (all *Ps* < 0.05). There were no significant differences in BMI, incision length, operative time or prosthesis type between fallers and non-fallers (all *Ps* > 0.05). Figure 2 shows balance-related outcomes between fallers and non-fallers. The AQoL and the ABC at T0 to T2 and the BBS at T0 and T2 were significantly lower in fallers than in non-fallers (all *Ps* < 0.05). In contrast, the FES-I and the TUG were significantly higher in fallers than in non-fallers at T0 and T2 (all *Ps* < 0.05). There were no significant differences in WOMAC (total and subscales) or knee extension strength between fallers and non-fallers (all *Ps* > 0.05).

**Table 3:** Comparisons of the general and surgical characteristics between fallers and non-fallers.

Parameters	T0		T1		T2	
	Fallers	Non-fallers	Fallers	Non-fallers	Fallers	Non-fallers
Patients (N)	56	320	20	301	11	339
Age (years)	73.79 ± 5.87*	68.08 ± 8.56	74.30 ± 3.16*	69.01 ± 8.08	71.27 ± 7.17	68.54 ± 8.60
Sex (male:female)	8:48*	109:211	2:18*	102:199	3:8	109:230
BMI (kg/m^2^)	26.69 ± 2.73	26.55 ± 2.79	26.73 ± 3.03	26.45 ± 2.74	27.00 ± 1.98	26.60 ± 2.82
KL grade of the contralateral knee	2.64 ± 1.09*	1.95 ± 0.75	2.55 ± 0.95*	1.95 ± 0.75	3.00 ± 0.98*	1.98 ± 0.77
Incision length (cm)			15.22 ± 0.81	15.52 ± 0.85	15.40 ± 0.70	15.54 ± 0.84
Operative time (min)			59.60 ± 5.92	60.23 ± 5.47	62.00 ± 5.73	60.38 ± 5.50
Prosthesis (mobile:fixed)			5:15	62:239	5:6	72:267

BMI, body mass index; T0, before surgery; T1, 1 year after surgery; T2, 2 years after surgery; KL, X-ray diagnostic criteria developed by Kellgren and Lawrence. *Compared with non-fallers, *P* < 0.05.

### Risk factors for falls before and after primary TKA

We performed binary logistic regression analysis of all factors, as shown in Table 3, to investigate the possible risk factors for falls before and after primary TKA. Table 4 shows that age (≥70 years), sex (female) and KL grade of the contralateral knee (≥3) were significant independent risk factors for a history of falling in the last preoperative year and the first postoperative year, and the latter was also a significant independent risk factor in the second postoperative year.

**Table 4:** Possible risk factors for falls before and after primary TKA.

Characteristics	T0 (N = 376)		T1 (N = 321)		T2 (N = 350)	
	N (fallers)	OR (95%CI)	N (fallers)	OR (95%CI)	N (fallers)	OR (95%CI)
Age						
<70 years	176 (7)	1.00	148 (1)	1.00	172 (3)	1.00
≥70 years	200 (49)	9.10 (3.83–21.61)*	173 (19)	21.46 (2.75–167.75)*	178 (8)	2.80 (0.68–11.51)
Sex						
Male	117 (9)	1.00	104 (2)	1.00	112 (3)	1.00
Female	259 (47)	3.18 (1.39–7.28)*	217 (18)	5.54 (1.17–26.20)*	238 (8)	1.65 (0.38–7.09)
Body mass index						
<25kg/m^2^	120 (15)	1.00	106 (6)	1.00	110 (2)	1.00
≥25 kg/m^2^	256 (41)	1.35 (0.66–2.76)	215 (14)	1.12 (0.38–3.33)	240 (9)	1.78 (0.35–9.14)
KL grade of contralateral knee						
<3	276 (24)	1.00	249 (9)	1.00	265 (2)	1.00
≥3	100 (32)	6.28 (3.24–12.19)*	72 (11)	6.54 (2.34–18.28)*	85 (9)	16.97 (3.45–83.64)*
Incision length						
<16 cm			208 (12)	1.00	221 (7)	1.00
≥16 cm			113 (6)	0.61 (0.21–1.82)	129 (4)	0.82 (0.21–3.17)
Operative time						
<60 min			153 (11)	1.00	159 (3)	1.00
≥60 min			168 (9)	0.77 (0.28–2.07)	191 (8)	2.47 (0.76–8.02)
Prosthesis type						
Mobile			67 (5)	1.00	77 (5)	1.00
Fixed			254 (15)	0.97 (0.29–3.24)	273 (6)	0.30 (0.08–1.13)

T0, before surgery; T1, 1 year after surgery; T2, 2 years after surgery; OR, odds ratios; CI, confidence intervals; KL, X-ray diagnostic criteria developed by Kellgren and Lawrence. *The results reached statistical significance, *P* < 0.05.

## Discussion

Knee OA is an established risk factor for falls, and TKA is the most frequently performed surgery to treat end-stage knee OA^32^. Therefore, the impact of TKA on the incidence of falls and balance-related outcomes in OA patients and the possible risk factors for falls before and after TKA are of considerable interest. This study found that the annual incidence of falls, AQoL, WOMAC (total and subscales), FES-I, ABC, knee extension strength, BBS and TUG improved significantly after primary TKA compared to preoperative values. Age (≥70 years), sex (female) and KL grade of the contralateral knee (≥3) were identified as significant independent risk factors for falls in the last preoperative year and the first postoperative year, and the latter was also an independent risk factor for falls in the second postoperative year after primary TKA.

The reported annual incidence of falls ranges from 11–34% in older Chinese community-dwelling people^2^, and the incidence is greater than 50% in people with knee OA and ranges from 7–40% in patients after TKA^5,7,8,18,19^. We reported a relatively lower incidence before (14.89%) and after primary TKA (6.23% and 3.14% in the first and second postoperative year, respectively) compared to the previous studies, which may be related to multiple factors, including fall-prevention strategies, data collection, and patient sources. Campbell *et al*. reported that single interventions were as effective in reducing falls as interventions with multiple components^33^, but Gillespie *et al*. reported that interventions aimed to improve knowledge on fall-prevention alone did not significantly reduce the risk of falls in older community-dwelling adults^7^. However, few studies compared the effects of interventions to improve fall-related knowledge on the incidence of falls between community-dwelling people and post-TKA patients. The fall-prevention strategies performed in this study included interventions to improve fall-related knowledge and functions. It is difficult to investigate whether increased fall-prevention knowledge alone affected the risk of falls after TKA. Fall-prevention strategies generally commence during the preoperative period and appear to be most effective when they target high-risk populations or individuals^33^. However, the optimal timing and interventions to prevent falls before and after TKA are rarely reported, and therefore, these factors are important areas for further study. Previous studies have also reported that fall recollection is commonly characterised by poor sensitivity compared to prospective fall tracking^34,35^, and the active tracking of falls after surgery may lead to reduced falls simply from participant awareness that their falls were being recorded (Hawthorne effect)^36^. A small number of patients in this study could not accurately recollect the details of the falls, especially before surgery, although most patients were very sure of the number and details of their falls (Table 2). It also possible that some patients were unable to recollect their falls despite not presenting any obvious memory dysfunction, and thus, the results may have underestimated the incidence of falls. The source of patients—approximately 70% of patients were from cities—may also have contributed to the low incidence of falls in the study; however, the effect of living environment on the incidence of falls must be further investigated.

Falls are the most frequent type of accident and the major cause of injury-related hospitalization in the elderly. Falls may cause older people to become fearful of falling even when falls do not result in physical injury, which reduces quality of life, restricts daily activities and results in the onset of functional decline^37^. Fear of falling is a common problem in people with lower limb arthritis and has serious consequences in relation to reduced activity and loss of confidence and independence^25^. Significant reduction in fear of falling and an increase in balance confidence were found after TKA compared to before TKA in this study, and this finding may be due, at least in part, to the considerable improvements in knee pain, stiffness and physical functions (WOMAC), which were consistent with previous studies^3,5,38^. Levinger *et al*. found that the AQoL was significantly reduced 4 months postoperatively compared to preoperative scores, which may be primarily associated with impairments in independent living and social relationships^5^. We found that the AQoL was slightly increased 1 year after surgery and remarkably improved 2 years after surgery, which indicates that the quality of life improved at least 1 year after primary TKA. However, self-reported measures alone may fail to capture actual changes in physical ability following TKA and overestimate performance changes^39–41^. Physical performance tests are necessary to further evaluate balance-related functions.

Deficits in muscular function are consistently reported in patients with knee OA and in patients who undergo knee arthroplasty^6,42–45^. Knee pain and quadriceps weakness affect the sensory and mechanical functions of the joint, and these factors are associated with increased postural sway^44–47^, which results in balance difficulties and increased risk of falls^48,49^. Several methods for assessing knee extension strength have been reported, such as direct measurement (Newton, pound and kilogram) and normalization to body mass and/or body height^5,50,51^. However, previous findings of altered knee extension strength following TKA are inconsistent and controversial, and there is no consensus on which method performs best^5,21,46,52,53^. In this study, the knee extension strength was normalized to the patient’s body height and body mass according to a previous study^5^ and was notably improved following primary TKA. There were no significant differences in normalized knee extension strength between fallers and non-fallers, but the fallers exhibited relatively weaker strength than non-fallers. BBS and TUG are commonly used physical performance tests for lower limb functions^29,54^, and these measurements are related to the risk of accidental falling in community dwelling adults^55,56^. Swinkels *et al*. reported increased BBS and reduced TUG (both improvements) 6 months after TKA compared to preoperative values, and the differences between pre- and postoperative TUG reached statistical significance^54^. We performed this study using a longer follow-up and found that BBS and TUG improved significantly 1 and 2 years after surgery compared to preoperative scores, which suggests that TKA improved the risk of accidental falling in patients with knee OA.

Various questionnaires and physical tests have been developed to assess balance-related functions^3,18^. However, these tests are not without limitations, and there is no consensus on which tool performs best. Therefore, a combination of different tools has been suggested^3,18^. Swinkels *et al*. reported that BBS was significantly negatively correlated with TUG and positively correlated with ABC, stiffness and physical functions of WOMAC before and after surgery. TUG was also significantly negatively correlated with ABC^54^. In contrast, Minzner *et al*. reported poor concurrent validity between patient-reported and performance-based measures of physical functions in patients who undergo unilateral TKA^57^. The present study also found that AQoL was positively correlated with BBS before surgery (*r* = 0.15, *P* < 0.01) and negatively correlated with TUG 1 year and 2 years after surgery (*r* = −0.13, *P* = 0.02; *r* = −0.13, *P* = 0.01, respectively) and that ABC was negatively correlated with TUG 1 year after surgery (*r* = −0.14, *P* = 0.01). Differences in the time assessments and the methods used may have affected these results, and further studies are needed to investigate the correlations between self-reported and physical performance outcomes for the risk for falls and balance-related functions.

Age and gender are important risk factors for falls, and older patients and women exhibit a higher likelihood of falling^37,58^. Our study found that age (≥70 years) and gender (female) were significant independent risk factors for falls in patients with end-stage OA and during the first postoperative year following primary TKA. A large proportion of patients who undergo TKA for the treatment of end-stage OA have concurrent OA in the contralateral knee^22^. Many studies demonstrated that pre- and postoperative factors in the contralateral knee, such as pain and knee extension strength^22,23^, were associated with knee functions after TKA, but few studies investigated the impact of contralateral knee OA status on falling. We found that the OA severity of the contralateral knee was an independent risk factor for falls before and after TKA, and KL grade ≥3 of the contralateral knee was associated with an increased likelihood of falling. Therefore, surgeons and physiotherapists should pay more attention to contralateral knee status, in addition to age and gender, in patients with knee OA before and after primary TKA when designing fall-prevention strategies.

Several limitations must be considered when interpreting the findings of this study. First, the participants in this study had primary and unilateral TKA due to OA. These results may only be generalized to individuals with similar characteristics, and further studies are warranted to expand the present results and the generalizability to patients with bilateral or unicompartmental knee arthroplasty or other conditions (such as RA). Second, fall data were collected using interviews or telephone calls, which primarily rely on the retrospective recall of falls by patients and may result in an underestimation of fall prevalence. Third, no non-surgical group with similar severity of knee OA was recruited as control, and all patients in this study received a similar postoperative rehabilitation programme. Thus, this study was unable to establish the natural course of falls and whether the postoperative rehabilitation accounted for the reduced incidence of falls. Fourth, although the strength testing data was normalized to body height and body mass, the lever arm at the point of force application was not taken into account, and therefore, the data were not fully normalized to subject anthropometrics. Finally, other factors, such as psychophysiological status, comorbidities and lifestyles were not documented and may have affected the results. However, to our knowledge, our findings reported the serial changes in the incidence of falls and balance-related outcomes and identified possible risk factors for falls before and after primary TKA in the largest population and for the longest period of follow-up of any existing study.

In conclusion, the data of this study suggest that primary TKA is associated with a reduced incidence of falls and improved balance-related functions in patients with OA. OA severity of the contralateral knee is a significant independent risk factor for falls, and surgeons and physiotherapists should pay more attention to the contralateral knee when designing fall-prevention strategies before and after primary TKA in patients with OA.
